# Association between depression, anemia and physical activity using isotemporal substitution analysis

**DOI:** 10.1186/s12889-023-17117-1

**Published:** 2023-11-13

**Authors:** Hee-kyoung Nam, Jungmi Park, Sung-il Cho

**Affiliations:** Department of Public Health Science, Graduate School of Public Health, Seoul National University, 1 Gwanak-ro, Gwanak-gu, Seoul, Republic of Korea

**Keywords:** Depression, Physical activity, Anemia, Isotemporal substitution model, KNHANES, GPAQ

## Abstract

**Background:**

Depression is a leading cause of disability and mortality, with estimated number of deaths exceeding 2.2 million worldwide. We examined depression in relation to anemia and physical activity, both of which have an impact on depression mechanisms.

**Methods:**

This cross-sectional study used data from the Korean National Health and Nutrition Examination Survey, including 18,622 participants. Depression was measured by The Patient Health Questionnaire-9, and physical activity was assessed by the Global Physical Activity Questionnaire. Anemia was defined by World Health Organization criteria for blood hemoglobin levels. Isotemporal substitution model for physical activity was used to assess the effect of replacing sedentary behavior to each intensity level of physical activity. Logistic regression was applied to estimate the association on depression.

**Results:**

Replacing sedentary behavior with moderate or vigorous physical activity was associated with a lower risk of depression in the anemic (OR: 0.875, 95% CI: 0.782–0.978) and non-anemic groups (OR: 0.943, 95% CI: 0.919–0.967). Depression risk was significantly reduced by replacing walking with moderate to vigorous physical activity in both anemic (OR: 0.877, 95% CI: 0.784–0.982) and non-anemic groups (OR: 0.951, 95% CI: 0.927–0.976).

**Conclusions:**

Moderate to vigorous physical activity had a protective association against depression in both anemic and non-anemic groups. Anemic patients are recommended to perform physical activity for any duration acceptable to them to prevent depression.

## Introduction

The estimated number of deaths from depression worldwide exceed 2.2 million [1]. In 2017, depressive disorders were among the most common causes of years lived with disability in females worldwide [2]. In Korea, a nationally representative survey found that the prevalence of depression was 11.3% in 2021 [3] and major depressive disorder was ranked as the tenth-leading cause of disease burden in 2018 [4]. Depression is a chronic disease that is affected by multiple social factors, including female sex, low socioeconomic status, food insecurity, and age [5]. Furthermore, depression has shown associations with other diseases such as anemia [6], stroke [7] and cardiovascular disease [8]. Given the significance of depression, it is necessary to examine the association between depression and other diseases.

For instance, it is important to pay attention to its relationship with anemia. Anemia prevalence of 2019 in Korea was 6.9% for total population; 2.6% for males and 11.5% for females [9]. Since the risk factors for anemia is various such as female sex, age, malnutrition, and infectious diseases [10–14], reducing the prevalence is challenging. In addition, the pathway of depression and anemia has been reported through recent studies. For example, one systematic review and meta-analysis reported that anemia may have negative effects on depression and the relationship was stronger in individuals aged ≥ 65 years [15]. Meanwhile, other study observed the significant association of anemia and depression in female group [16].

The relationship between depression and anemia appears to involve various mechanisms working in a complex manner and has been previously explained in elsewhere [15, 16]; First, a lower hemoglobin level has been associated with high regional cerebral blood flow [17], which in turn can lead to being in depressive mood [18]. Second, low hemoglobin can cause lack of oxygen for the body tissue and it diminishes the capacity of physical activity, which then leads to decreased mental health. For instance, people with anemia become tired easily when they perform physical activity, and are likely to be short of breath. Third, by worsening the physical and mental condition, prolonged symptoms of anemia are associated with the indicators that could potentially contribute to the onset of depression [19]. For example, due to the main symptoms of anemia such as fatigue [20], anemic population showed the low self-rated health [21] and low quality of life [22]. Based on the clinical mechanism described above, individuals with anemia require careful surveillance to prevent the development of depression.

In such situation, physical activity can play a role as an intervention to reduce the risk of depression [23, 24]. Following the World Health Organization (WHO) standard [25, 26], it is officially recommended to perform 150–300 min of moderate physical activity (MPA), or 75–150 min of vigorous physical activity (VPA); or an equivalent combination of moderate to vigorous physical activity (MVPA) a week, and to minimize time spent with inactive or sedentary behavior (SB). Scientific evidence has supported the health benefits of engaging in physical activity for addressing depression [26, 27] with underlying biological and psychological mechanisms. First, physical activity plays an antidepressant role by enhancing the circulation of neurotrophic factors. Second, it increases the secretion of antioxidant factors, potentially reducing the risk of depression. Third, physical activity reduces psychological stress by promoting social participation and increases self-esteem. However, the specific health benefits of physical activity vary depending on its type, such as VPA, MPA, and walking.

When considering the health benefits of physical activity on depression, we hypothesized that anemic patients may be encouraged to perform physical activity to prevent from depression. Regardless of the symptom of anemia, necessity to perform a physical activity is still existing. According to one study, physical activity had a positive association on the reduced symptoms of anemia [28]. Additionally, individuals with anemia might be more susceptible to displaying increased SB as a result of their symptoms. Considering this, it becomes crucial to monitor the time spent engaging in physical activities and SB that can affect the progression of depression. However, as far as our current knowledge extends, an official physical activity guideline for the anemic population has not yet been established, and there exists a knowledge gap regarding the association of physical activity intervention on the progression to depression in anemic population.

Thus, we explored the relationship between depression and physical activity in an anemic population in this study. The association of depression and physical activity on anemic population is needs to be considered when refer to the current prevalence of anemia and depression. Also, it is important to perform the prompt extent of physical activity for the depressive anemic population. Research has been conducted to explore the potential health effects of engaging in physical activity below recommended levels [29], and according to recent studies, it has been revealed that engaging in physical activity, even for durations below the recommended 10 min, confers health benefits [30]. Therefore, even though the time is below the recommended duration for physical activity, in this study, we set a suitable time of 5 min as the alternative timeframe to observe substitution association.

To propose practical and suitable time frames for physical activity among anemic population, we employed the isotemporal substitution method (ISM). Since the introduction of ISM, many researches have been conducted using this methodology. For example, one study found the significant association of lower depressive symptoms and physical activity when substituting an hour of SB to VPA [31]. Other research observed the association of lower depression risk when substitute an hour of television watching to brisk walking [32]. Hence, when weighing the adverse effects of sedentary behavior against the health advantages of physical activity at various intensities, it becomes essential to proactively consider the versatility of the ISM, which hinges on the concept of substitution.

In this study, we estimated substitutional association of types of physical activity and depression in anemia population, when physical activity is replaced by sedentary behavior by using Korean National Health and Nutrition Examination Surveys (KNHANES).

## Methods

We utilized four KNHANES surveys (2014, 2016, 2018, and 2020) conducted by the Korea Disease Control and Prevention Agency (KDCA) [33–35]. KNHANES is a nationwide cross-sectional investigation that calculates representative, reliable national statistics to measure health behavior, physical condition, and nutritional status [36] and the survey is conducted annually. KNHANES uses proportional stratified sampling of Korean nationals, considering the region, districts and types of housing such as living in apartment or single-family house. However, survey was limited to 180 stratified sampling due to the Covid-19, which was originally conducted with 192 stratified sampling in other years. For example, the seventh KNHANES examined 192 survey clusters, 4416 households, and 10,000 individuals as a national sample. KNHANES conducts the survey throughout the year; January to December. This study included KNHANES from years when the Patient Health Questionnaire-9 (PHQ-9) was administered in 2014, 2016, 2018 and 2020. Of the 31,051 participants in these 4 years, those aged < 19 years (n = 6071) or with missing values/no response/not included (n = 6358) were excluded, leaving 18,622 participants. This study was approved by the Institutional Review Board of Seoul National University (no. E2009/003–013).

### Depression

Depression status was estimated using the PHQ-9 administered by trained health personnel. The validity and reliability of the Korean version of PHQ-9 has been verified [37]. The questionnaire consists of nine questions. Each has four answering options: not at all, several days, more than half the days, and nearly every day. On summing the answers, the depression level is classified into five groups: minimal (0–4), mild (5–9), moderate (10–14), moderately severe (15–19), or severe (20–27) [38]. Participants with more than mild depression (score > 5) were considered depressed, which was changed to a binomial outcome variable for analysis.

### Physical activity

The physical activity questions of KNHANES are in the Global Physical Activity Questionnaire (GPAQ), which includes four sections: Activity at work, Travel to and from places, Recreational activities, and Sedentary behavior. The questions ask how much time is spent on different types of physical activity, such as VPA, MPA, and inactive behavior such as SB [39]. Here, we focused on recreational physical activities and SB, and did not consider the travel to and from places, because our study examined the effect of recreational physical activity. A rest domain, i.e., activity at work, was adjusted in the model. To detect the effect of light physical activity, we considered the independent walking questionnaire of KNHANES, which was similarly designed to evaluate VPA and MPA in the GPAQ. VPA was defined as activity that increased breathing and heart rates, such as running, climbing a mountain, basketball, swimming, playing badminton, or jumping rope. MPA was limited to activities that caused a small increase in breathing or heart rate, such as brisk walking, jogging, playing golf, dancing, weight training, or Pilates. Walking includes the activities of walking from work or school with the intent of physical activity. VPA, MPA, and walking were considered only if the activity was continued for at least 10 min, as specified in the GPAQ. The exploratory variables VPA and MPA were summed as MVPA, similar to previous studies [40, 41]. The definition of SB considered how much time was spent sitting or reclining per day, such as sitting at a desk, sitting with friends, traveling in a car, bus, or train; reading; playing cards; or watching television; however, time spent sleeping was excluded.

For the descriptive analysis, we determined the participation rate in physical activity using the standard formula, which calculates the percentage of individuals engaging in either at least 2 hours and 30 minutes of MPA per week, at least 1 hour and 15 minutes of VPA per week, or a combination of both (with 1 minute of VPA considered equivalent to 2 minutes of MPA). Also, in Tables 2, the physical activity variables were defined as binomial variables, grouping the answers as yes or no according to the physical activity performance status following the GPAQ design. They were also used as continuous variables by recalculating all the time units in minutes for convenience in ISM in Tables 3 and 4 For participants who answered ‘no’ to engaging in physical activity, their response was recorded as 0 min. For those who answered ‘yes’ to performing physical activity, we calculated their total minutes by multiplying the frequency (days) by the hours and minutes spent. We then divided this total by 7 days to estimate the daily minutes of physical activity. In the ISM model, the standard of substitution was set to 5 min, because it was the best value when considering the mean time each physical activity was performed a day.

### Anemia

KNHANES determined the hemoglobin (HB), hematocrit, red blood cell count, and white blood cell count from blood sampling as a biomarker of anemia for the participants who agreed. Blood was sampled by a trained worker following the KNHANES manual. Before drawing the blood, the health personnel must ask participants when they last ate and record their answers. In this study, anemia was defined by the HB level, considering sex and age, following the World Health Organization criteria [42]. Males aged > 15 years with HB < 13 g/dL, pregnant females aged > 15 years with HB < 11 g/dL, and non-pregnant females aged > 15 years with HB < 12 g/dL were considered to have anemia. In addition, the variables were used as binomial explanatory variable.

### Covariates and study design

Socioeconomic variables were evaluated, including sex, age, marital status, occupation, smoking, and BMI. Sex was divided into males and females according to the participant responses. Age was classified into three categories: 19–39, 40–59, ≥ 60 years. Marital status was divided into ever married, including separated, divorced or widowed, and never married. Occupation was separated into four groups based on similarities among the work characteristics, including white collar (managers and experts), pink collar (service or sales workers), blue collar (agricultural and fisheries workers or simple laborer), and grey collar (housewife, unemployed, or student). In addition, we considered the four individual years as a dummy for analysis. Smoking status was divided into current smoker, former smoker, and non-smoker. BMI was categorized into normal, low weight, and overweight.

This was a cross-sectional study, considering that the KNHANES is an annual population-based investigation implemented within a survey year. We constructed the dataset from four individual years and analyzed the association of depression and physical activity in the anemic group.

### Isotemporal substitution method

**Fig. 1:**
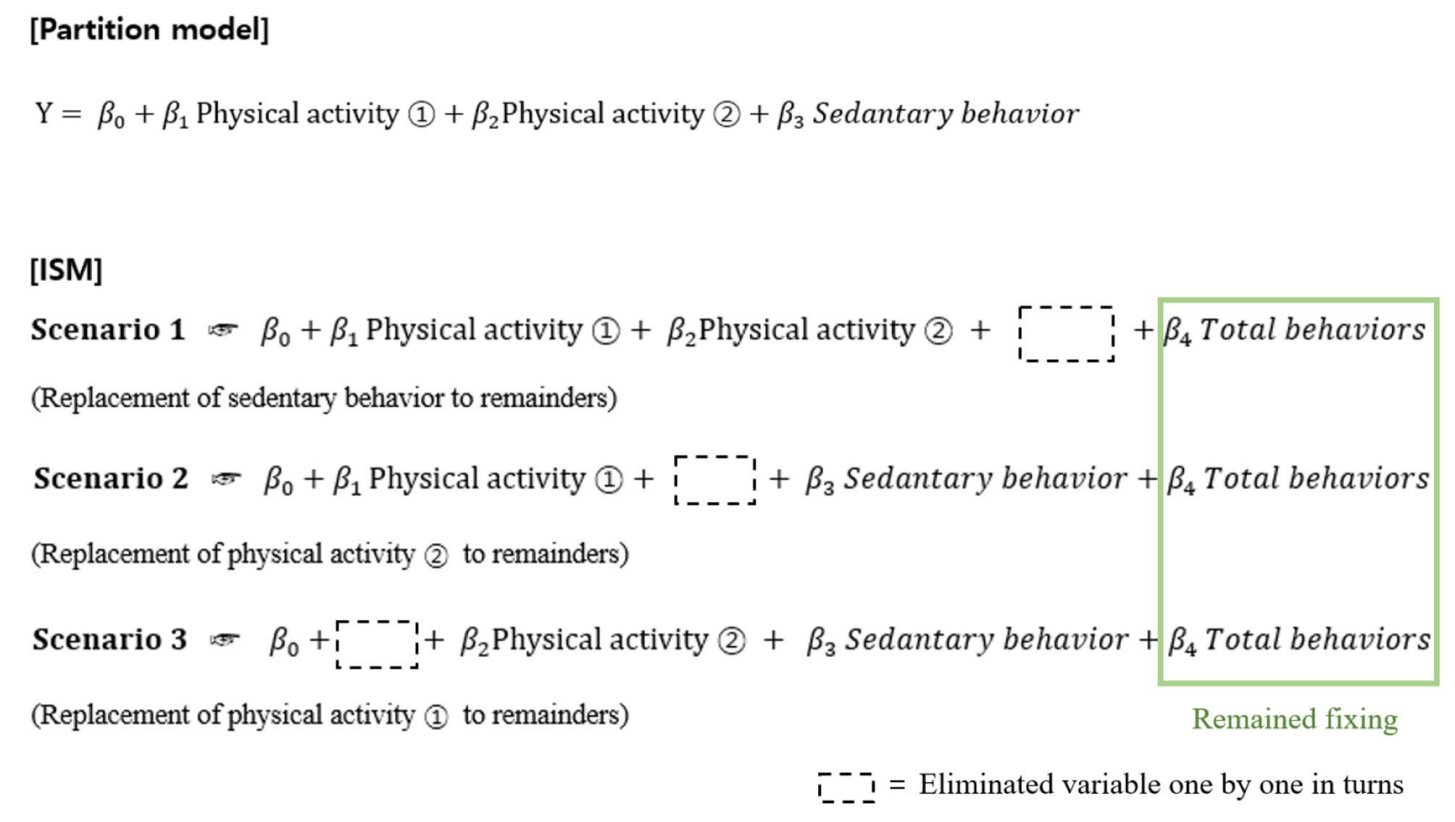
Model configuration diagram of ISM compared to a partition model

The ISM was introduced in 2009 [43] and strengthened during substitution. Given the 24 h day length limitation, the ISM assesses the effect when a specific activity is replaced by another activity of interest. The methodology derives its strength from substitutional concepts, particularly as physical activity is linked to reduced health risks. Numerous studies have been undertaken to replace time spent in SB with physical activity, which is associated with a heightened likelihood of positive health outcomes. This study examined the substitution effect of physical activity in an anemic group according to its intensity, while fixing depression as the outcome variable. ISM includes three different multiple linear regression models [43]. First, the substitution model is based on the time replacement concept. This model uses all physical activity variables, SB and total behavior time. Total behavior time which defined as the sum of MVPA, walking, and SB is required to observe the estimates of the association when one physical activity is substituted for another. These substitution results are manifested through a process in which variable total behavior time is consistently included while the remaining variables are successively removed by turns (Fig. 1). Second, the partition model detects different association with the outcome variable of one physical activity while fixing the remaining activities. We included MVPA, walking, and SB in the partition models. This model does not represent the time replacement effect, because the total behavior time is not considered a variable. Finally, the single model uses one physical activity with each of the other covariates. For this single model, we included types of physical activity, SB and total behavior time in turns.

### Statistical analysis

First, the depression distribution was determined using the chi-square test to suggest a descriptive characteristic of the participants. Second, to observe the substituted effect of each of the physical activity variables, we used the isotemporal substitution approach, which involves adding and deleting each of the variables in turn in the model. Two-sided *P* values were used to determine statistical significance and odds ratios (OR) with 95% confidence intervals (CI) were both provided. All analyses were conducted with SAS 9.4 (SAS Institute, Cary, NC, USA) using the SURVEY FREQ, SURVEY MEANS, and SURVEY LOGISTIC procedures.

## Results

### Descriptive trends and characteristics of the participants

**Fig. 2:**
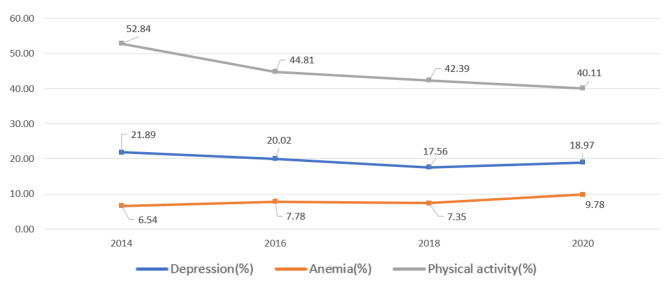
Prevalence of depression, anemia and physical activity* among study participants (2014–2020). *Physical activity prevalence was defined by the proportion of population meeting WHO recommendations (150–300 min of moderate physical activity (MPA), or 75–150 min of vigorous physical activity (VPA), or an equivalent combination of moderate to vigorous physical activity (MVPA) per week)

Fig. 2 illustrates the changing prevalence rates of depression and anemia over the years (2014–2020) among the study participants. Depression showed a steady decline until 2018 but began to rebound in 2020. Conversely, the prevalence of anemia consistently increased from 6.54–9.78%. Meanwhile, the participation rate in physical activity exhibited a continuous downward trend from 2014 to 2020.

Depression was present in 21.65% of anemic participants versus 19.27% of non-anemic participants (Table 1). Depression showed increasing trends in both anemic group and non-anemic group. Sex, age, marital status, and smoking status were significantly different between depressed and non-depressed individuals in the anemic and non-anemic groups. However, occupation and BMI were significantly different in the non-anemic group only.

**Table 1:** Descriptive characteristic of the respondents by key variables (n = 18,622)

	Anemic group (n = 1704)				Non-anemic group (n = 16,918)			
	Depression (n [%])				Depression (n [%])			
	N	%	Total (N/%)	*P-value*	N	%	Total(N/%)	*P-value*
Total	370	21.65	1704 (100)	< 0.001	3248	19.27	16,918 (100)	< 0.001
Year								
2014	73	4.46	294 (17.17)	0.213	759	4.57	3513 (21.16)	0.003
2016	103	5.71	452 (25.01)		908	5.05	4493 (25.56)	
2018	99	5.36	436 (25.05)		818	4.69	4750 (27.21)	
2020	95	6.10	522 (32.75)		763	4.95	4162 (26.05)	
Sex								
Male	54	2.45	378 (19.60)	< 0.0001	1070	7.47	7521 (50.95)	< 0.001
Female	316	19.20	1326 (80.39)		2178	11.80	9397 (49.04)	
Age								
19–39	88	7.04	252 (26.03)	< 0.001	1154	8.63	4986 (37.18)	< 0.001
40–59	94	6.19	421 (40.56)		1108	6.92	6477 (41.44)	
60≤	188	8.42	509 (33.39)		986	3.71	5455 (21.36)	
Marital status								
Yes	321	17.10	1542 (87.62)	< 0.001	2494	13.32	13,895 (75.62)	< 0.001
No	49	4.55	162 (12.37)		754	5.94	3023 (24.37)	
Occupation								
White	53	4.09	340 (23.06)	0.075	686	4.60	4279 (28.39)	< 0.001
Pink	42	2.83	201 (13.25)		472	3.04	2356 (24.76)	
Blue	55	3.01	297 (16.47)		578	3.35	4038 (23.29)	
Grey	220	11.71	866 (47.19)		1512	8.27	6245 (33.54)	
Smoking status								
Never	282	16.95	1289 (77.42)	0.038	1960	10.76	10,107 (55.88)	< 0.001
Former smoker	52	2.69	295 (15.89)		566	3.42	3585 (21.59)	
Current smoker	36	2.00	120 (6.67)		722	5.08	3226 (22.52)	
BMI (kg/m^2^)								
< 18.5	24	1.55	114 (6.93)	0.377	206	1.31	680 (4.29)	< 0.001
18.5–25	239	13.62	1141 (66.63)		1925	11.35	10,203 (59.83)	
> 25	107	6.48	449 (26.43)		1117	6.60	6035 (35.87)	
25+	81 (6.10)	240 (18.36)	321 (24.46)	(21.87–27.05)	895 (6.39)	3,989 (28.43)	4,884 (34.82)	(33.77–35.87) 35.8655

### Association of physical activity, depression, and anemia

Table 2 presents the descriptive statistics of physical activity status according to the depression and anemia status. The descriptive results in Table 2 were initially presented as the number of participants (%) engaging in physical activity less than or more than 10 min per day, respectively. This classification was made according to the criteria of the questionnaire composition of GPAQ. In terms of SB, since GPAQ does not divide based on a criterion on over or under 10 min, this distinction was unnecessary. In addition, mean ± standard deviation and median has been suggested for physical activity and SB. A higher proportion of individuals performed VPA in the non-anemic group (9.04%) than in the anemic group (3.90%), which is in line with the trends observed in the depressed group. Similarly, a higher proportion of individuals performed MPA in the non-anemic group (24.80%) than in the anemic group (17.39%). In addition, the mean VPA durations were lower for the anemic group (0.63 min/day) than for the non-anemic group (0.87 min/day). MPA durations were also show the similar mean pattern; 1.72 min/day for anemic group, and 3.55 min/day for non-anemic group. Non-anemic participants (82.95%) had a higher walking percentage compared to anemic participants (79.91%). Also, the mean walking time was longer for the non-anemic group (46.15 min/day) than for the anemic group (44.36 min/day). However, anemia with depression group showed higher SB duration in means (524.66 min/day) and median (521.21 min/day) compare to non-anemia group; mean: 524.66 (min/day) and median: 479.91 (mins/day).

**Table 2:** Type of physical activity and SB by anemia and depression status

		Anemic group (n = 1704)				Non-anemic group (n = 16,918)			
		Depression				Depression			
		Yes (n = 370)	%	Total (N/%)	*P-value*	Yes (n = 3248)	%	Total (N/%)	*P-value*
VPA (min/day)	< 10	355	94.46	1643 (96.10)	< 0.001	3041	92.06	15,656 (90.96)	< 0.001
	≥ 10	15	5.53	61 (3.90)	0.0018	207	7.93	1262 (9.04)	< 0.001
	mean	0.63		0.49		0.87		1.03	
	median	0		0		0		0	
	SD	2.45		2.60		3.02		3.30	
MPA (min/day)	< 10	334	89.11	1438 (82.61)	< 0.001	2671	79.41	13,061 (75.20)	< 0.001
	≥ 10	36	10.88	266 (17.39)	< 0.001	577	20.58	3857 (24.80)	< 0.001
	mean	1.74		2.98		3.55		4.37	
	median	0		0		0		0	
	SD	5.77		7.45		8.12		9.16	
Walking (min/day)	< 10	101	24.96	374 (20.08)	< 0.001	690	19.08	3127 (17.05)	< 0.001
	≥ 10	269	75.03	1330 (79.91)	< 0.001	2558	80.91	13,791 (82.95)	< 0.001
	mean	44.36		44.65		46.15		48.82	
	median	27.54		28.20		29.66		29.78	
	SD	51.53		50.96		54.52		55.54	
SB (min/day)	mean	524.66		494.60		523.27		492.54	
	median	521.21		465.52		479.91		476.87	
	SD	245.55		221.46		230.69		217.90	

* VPA: vigorous physical activity; MPA: moderate physical activity; SB: sedentary behavior

* The classification of less than 10 min was based on the questionnaire composition criteria of GPAQ. Participants who reported engaging in each physical activity for less than 10 min were considered as having not performed physical activity

### Isotemporal substitution model for physical activity and SB

Tables 3 and 4 present the results of ISM in the anemic and non-anemic groups, respectively. Three types of model were analyzed for each group. The ISM model considered the time replacement effect of each of the physical activities and SB. We observed the time replacement effect at 5 min, based on the daily time spent in physical activities by the study participants, which demonstrated the daily substitution effect of one activity on the other. In the model, (a) reveals the odds of having depression after substituting 5 min of SB for 5 min of walking or MVPA, respectively. (b) and (c) share the same interpretation logic. For the anemic participants, replacing 5 min of SB by 5 min of walking decreased the odds of having depression, albeit without statistical significance. On the other hand, when SB was substituted by MVPA (OR: 0.875, 95% CI: 0.782–0.978), the risk of depression was significantly decreased. Furthermore, replacing walking with SB was associated with statistically non-significant higher odds of having depression. Replacement of walking with MVPA was associated with decreased odds of having depression (OR: 0.877, 95% CI: 0.784–0.982). Interestingly, when 5 min of MVPA were substituted by 5 min of SB (OR: 1.143, 95% CI: 1.022–1.279) and walking (OR: 1.140, 95% CI: 1.018–1.276), the odds of having depression were significantly increased. In addition, only MVPA was associated with decreased odds of having depression in the single model (OR: 0.885, 95% CI: 0.790–0.992) and partition model (OR: 0.886, 95% CI: 0.792–0.991).

**Table 3:** Time replacement effect of physical activity and the other models in anemic participants (n = 1704)

	(5 min/day)^***^	SB^*^	Walking	MVPA^*^
Isotemporal substitution model (ISM)^**^	Substitute SB (a)	Replaced	0.997 (0.982–1.012)	**0.875** **(0.782–0.978)**
	Substitute walking (b)	1.003 (0.988–1.018)	Replaced	**0.877** **(0.784–0.982)**
	Substitute MVPA (c)	**1.143** **(1.022–1.279)**	**1.140** **(1.018–1.276)**	Replaced
Single model^**^	Single	1.003 (1.000–1.006)	0.998 (0.983–1.013)	**0.885** **(0.790–0.992)**
Partition model^**^	Partition	1.003 (1.000–1006)	1.000 (0.985–1.015)	**0.886** **(0.792–0.991)**

* SB: sedentary behavior; MVPA: moderate to vigorous physical activity

** ISM was adjusted for sex, age, marital status, year, sleep, and occupational physical activity and total behavior time. Single model was adjusted for sex, age, marital status, and year. Partition model was adjusted for sex, age, marital status, year, and other types of physical activity

(a) SB was replaced by physical activities

(b) Walking was replaced by SB and MVPA

(c) MVPA was replaced by SB and walking

*** This calculation involved multiplying the frequency (days), hours (per day), and minutes (per day), which were then divided by 7 days and converted into 5 min units

For the non-anemic participants, replacing 5 min of SB by walking (OR: 0.991, 95% CI: 0.986–0.996) or MVPA (OR: 0.943, 95% CI: 0.919–0.967) significantly decreased the risk of depression. However, the odds of having depression were increased when 5 min of walking were replaced by SB (OR: 1.009, 95% CI: 1.004–1.014). Furthermore, the odds of having depression were decreased when walking was substituted by MVPA (OR: 0.951, 95% CI: 0.927–0.976). Replacing MVPA with SB (OR: 1.060, 95% CI: 1.034–1.088) or walking (OR: 1.051, 95% CI: 1.024–1.079) was associated with increased odds of having depression. In the single and partition models, MVPA significantly decreased the odds of having depression, whereas SB increased the odds of having depression.

**Table 4:** Time replacement effect of physical activity and the other models for non-anemic participants (n = 16,918)

	(5 min/day)^***^	SB^*^	Walking	MVPA^*^
Isotemporal substitution model (ISM)^**^	Substitute SB (a)	Replaced	**0.991** **(0.986–0.996)**	**0.943** **(0.919–0.967)**
	Substitute walking (b)	**1.009** **(1.004–1.014)**	Replaced	**0.951** **(0.927–0.976)**
	Substitute MVPA (c)	**1.060** **(1.034–1.088)**	**1.051** **(1.024–1.079)**	Replaced
Single model^**^	Single	**1.003** **(1.002–1.005)**	**0.994** **(0.989–0.999)**	**0.946** **(0.922–0.970)**
Partition model^**^	Partition	**1.003** **(1.002–1.005)**	0.996 (0.991–1.000)	**0.947** **(0.924–0.971)**

* SB: sedentary behavior; MVPA: moderate to vigorous physical activity

** ISM was adjusted for sex, age, marital status, year, sleep, and occupational physical activity and total behavior time. Single model was adjusted for sex, age, marital status, and year. Partition model was adjusted for sex, age, marital status, year, and other types of physical activity

(a) SB was replaced by physical activities

(b) Walking was replaced by SB and MVPA

(c) MVPA was replaced by SB and walking

*** This calculation involved multiplying the frequency (days), hours (per day), and minutes (per day), which were then divided by 7 days and converted into 5 min units

## Discussion

We investigated the protective association of physical activity against depression based on anemic status. Despite their association, a comprehensive approach has not been reported. According to the descriptive results, among depressive participants, those with anemia shows lower time duration for all types of physical activity while they spent longer time for SB. Then multiple linear regression results showed that physical activity had a protective role in anemic patients. MVPA was associated with decreased odds of having depression in anemic patients, and the substitution association by ISM was also observed when walking and SB were replaced by MVPA in the anemic and non-anemic groups.

Our results highlight the protective effect of substitution of SB with physical activity on depression in the anemic group. Fatigue and weakness may prevent anemic individuals from participating in physical activity. Nevertheless, the risk of depression was reduced when SB and walking were substituted by MVPA. Considering the low health-related quality of life [44] and increasing trend of anemia, prompt public health interventions for anemic population are necessary, such as developing physical activity guidelines. Although physical activity guidelines are available for the general population and patients with chronic diseases, they do not make recommendations for individuals with anemia [26, 45].

We used 5 min as the unit of substitution in ISM for 2 reasons. First, 5 min duration was the most practical and relevant choice for the anemic population, considering their reduced physical activity tendencies and the constraints posed by their symptoms on engaging in physical activity. Second, based on recent findings, health benefits can be obtained from engaging in physical activity even with a short duration, a relevance that extends to populations with anemia. For instance, a systematic review suggests that any duration of physical activity still yields positive health benefits on diverse health outcomes and overall mortality [30].

Our results for general population without anemia are in line with those of previous studies, which showed that physical activity has protective effects against depression. A survey conducted by the World Health Organization showed that lower VPA is associated with a higher rate of depression among middle-aged and older individuals [46]. In addition, a European study demonstrated that participation in VPA and MVPA at least once per week reduced the risk of depression [47, 48]. A study from the United States also demonstrated an association between MVPA and decreased risk of depression [49]. In addition, a recent Korean study demonstrated a notable decrease in the odds ratio of experiencing depression among participants who engaged in recreational physical activities, including VPA and MPA. However, there was a lack of data regarding depression and physical activity within the anemic group.

Furthermore, our results extend previous evidence regarding the association between physical activity and depression by considering the interaction with anemia. We observed a shorter duration of physical activity at all intensity levels in anemic individuals than in non-anemic individuals, probably due to the symptoms of anemia. However, anemic patients who participated in MVPA had a reduced risk of depression. These findings were also supported by the ISM approach. Although previous studies have demonstrated health benefits, such as lower risk of depression, and improved subjective health by the substitution of SB with physical activity [32, 50], the results were not stratified by anemia status. Our ISM results showed that substitution of SB with physical activity was associated with a significantly lower risk of depression in both anemic and non-anemic groups.

Our results will contribute to the development of physical activity recommendations for anemic patients. At least 150–300 min of MPA, 75–150 min of VPA, or equivalent combination of MVPA per week is recommended for all adults [25, 51, 52]. However, patients with anemia may not be able to accomplish the recommended time or intensity threshold of physical activity. Nevertheless, our findings suggest that the benefit of physical activity in preventing depression among anemic patients exists without a specific threshold in a dose-response fashion. Health personnel should recommend physical activity to patients with depression and anemia for any duration acceptable to them. Further studies are needed to stratify depression and anemia by severity in a larger population.

This study had several limitations. First, we did not perform subgroup analyses by age due to a small sample size. Physical activity recommendations differ by age and life course; therefore, subgroup analyses are recommended for better clinical application. However, we adjusted all models for age. Second, we could not exclude potential effects of pandemic in 2020 in our analysis. Recent studies observed that physical activity was reduced, and depression was increased due to the pandemic [53, 54]. These changes might have resulted in an overestimation of the association between physical activity and depression in 2020. However, we adjusted for the years in our model, and there was no significant interaction between the year and physical activity in association with depression. Therefore, we consider our results would not have been substantially biased by the effects of pandemic. Third, we determined physical activity levels based on self-reported answers to questionnaires, rather than using objective accelerometer recordings. It is unclear whether subjective recall of physical activity varies by anemia status. The use of an accelerometer in future studies may support our findings. Fourth, we did not explore the potential adverse effects of excessive physical activity in anemic patients, which should be investigated in future studies to establish the optimum range of physical activity for individuals with anemia. Lastly, due to the cross-sectional design of our study, there is a lack of a causal relationship for interpretation.

## Conclusion

Physical activity had significant protective association against depression in anemic individuals. MVPA and replacement of other types of physical activity by MVPA are associated with a decreased risk of depression. Anemic patients are recommended to perform physical activity for any duration acceptable to them to prevent depression.

## Data Availability

The datasets utilized in the present study can be accessed publicly on the official website of the KDCA at https://knhanes.kdca.go.kr/knhanes/eng/index.do.
